# Exosomes as a new frontier of cancer liquid biopsy

**DOI:** 10.1186/s12943-022-01509-9

**Published:** 2022-02-18

**Authors:** Dan Yu, Yixin Li, Maoye Wang, Jianmei Gu, Wenrong Xu, Hui Cai, Xinjian Fang, Xu Zhang

**Affiliations:** 1 Jiangsu Key Laboratory of Medical Science and Laboratory Medicine, School of Medicine, Jiangsu University, Zhenjiang, 212013 Jiangsu China; 2 Department of Clinical Laboratory Medicine, Nantong Tumor Hospital, Nantong, 226361 Jiangsu China; 3 Key Laboratory of Molecular Diagnostics and Precision Medicine for Surgical Oncology in Gansu Province, Gansu Hospital of Jiangsu University, Lanzhou, 730000 Gansu China; 4 Department of Oncology, Lianyungang Hospital Affiliated to Jiangsu University, Lianyungang, 222000 Jiangsu China

**Keywords:** Exosome, Cancer, Liquid biopsy, Biomarker, Precision medicine

## Abstract

Liquid biopsy, characterized by minimally invasive detection through biofluids such as blood, saliva, and urine, has emerged as a revolutionary strategy for cancer diagnosis and prognosis prediction. Exosomes are a subset of extracellular vesicles (EVs) that shuttle molecular cargoes from donor cells to recipient cells and play a crucial role in mediating intercellular communication. Increasing studies suggest that exosomes have a great promise to serve as novel biomarkers in liquid biopsy, since large quantities of exosomes are enriched in body fluids and are involved in numerous physiological and pathological processes. However, the further clinical application of exosomes has been greatly restrained by the lack of high-quality separation and component analysis methods. This review aims to provide a comprehensive overview on the conventional and novel technologies for exosome isolation, characterization and content detection. Additionally, the roles of exosomes serving as potential biomarkers in liquid biopsy for the diagnosis, treatment monitoring, and prognosis prediction of cancer are summarized. Finally, the prospects and challenges of applying exosome-based liquid biopsy to precision medicine are evaluated.

## Introduction

**Fig. 1:**
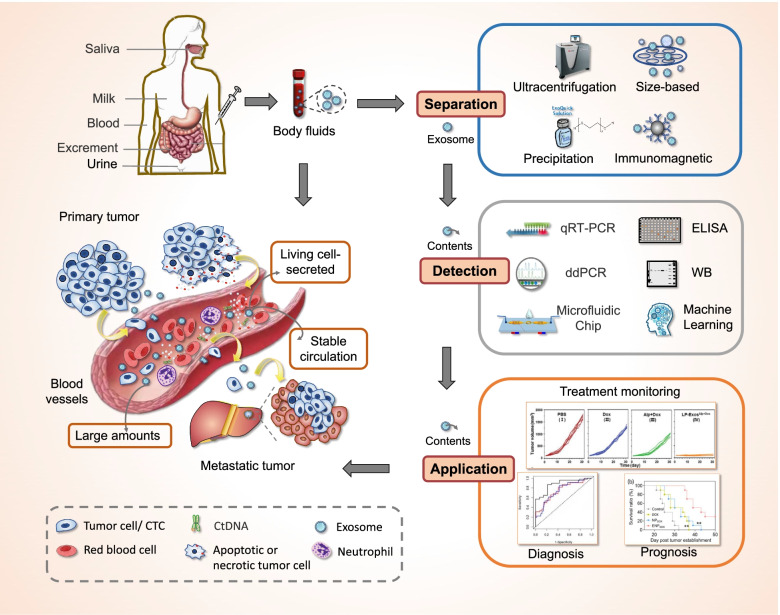
Exosomes as a new target for liquid biopsy. Exosomes are enriched in body fluids and are critically involved in tumorigenesis, tumor progression and metastasis. Compared with CTC and ctDNA, exosomes show superior characteristics such as living-cell secreted vesicles, large amounts and stable circulation. Traditional and advanced technologies have been used to separate exosomes from various body fluids and to detect exosomal cargoes. The detection of specific molecules of exosome may provide a new strategy for cancer diagnosis, progression monitoring, and prognosis prediction

Cancer is a leading cause of death around the world [1]. Mounting evidence suggests that the development of cancer is a dynamic process and diverse components are involved, including tumor cells, stromal cells, and immune cells. Up to now, tissue biopsy has been considered as the most common method for cancer diagnosis [2]. However, the extracted small tissues fail to represent tumor heterogeneity or monitor dynamic tumor progression, and the potential of metastasis may be increased by this invasive method, finally leading to poor survival and prognosis [3]. Due to minimal invasion, liquid biopsy, which collects the specimen of biofluids such as blood and urine, has drawn widespread attention and generated more opportunities for cancer diagnosis as well as real-time monitoring [4]. Exosomes, with a diameter of 40-160 nm, are lipid bi-layer membrane vesicles that are actively released by most cells and stably circulate in body fluids [5, 6]. Originally underestimated as vehicles for disposal of cellular waste products, exosomes are now being recognized as important players in intercellular communication [4, 7]. Accumulating evidence suggests that a variety of bioactive molecules, including nucleic acids, proteins, and lipids, are enriched in exosomes and could be transferred from donor cells to recipient cells, leading to the intracellular transfer of information [4, 8–10]. The bioactive cargoes in exosomes may be uptaken by recipient cells, facilitating tumorigenesis and tumor progression. In addition, exosomes are involved in the formation of pre-metastatic niche, tumor angiogenesis, and tumor immune suppression. Moreover, exosomes could reflect the altered physiological and pathological state of their parental cells [11–14]. These findings have led to the idea that analyzing the circulating exosomes and their derived cargoes may provide new opportunities for cancer liquid biopsy (Fig. 1), highlighting the potential of exosomes as biomarkers for cancer diagnosis, progression monitoring, and prognosis prediction.

Currently, circulating tumor cells (CTCs), circulating tumor DNA (ctDNA) and exosomes have become the three main branches of liquid biopsy [15, 16]. Compared with CTCs and ctDNA, exosomes have shown greater advantages in liquid biopsy. First, the presence of large amounts of exosomes (~ 10^9^ particles/mL) in biofluids contributes to relatively easy obtaining of vesicles, while only several CTCs exist in 1 mL blood samples [14]. Second, exosomes are secreted by living cells and inherent abundant biological information from their parental cells. Therefore, exosome is more representative than ctDNA, which limitedly reflects the information of apoptotic or dead tumor cells [11, 14]. Third, exosomes are innately stable because of their lipid bilayers, and thus stably circulate in physiological conditions even in harsh tumor microenvironment. The high biological stability allows long-term storage of specimens for exosome isolation and detection [17]. Notably, one of the big challenges for the application of exosomes in liquid biopsy is isolation with high efficiency and purity, which arises from their nanoscale size and intrinsic heterogeneity [18–20]. Moreover, since cancerous exosomes represent only a small fraction of all exosomes present in body fluids, ultrasensitive and specific detection is a prerequisite for the development of exosome-based cancer diagnostics. To date, a variety of methods have been developed for exosome isolation as well as the detection of exosomal proteins and nucleic acids [21–26]. Although notable progress has been made, the limited sensitivity and specificity, low purity and throughput remain significant challenges for academic research and practical use [27]. Therefore, active research is needed to develop an easy-to-operate, high-sensitivity and high-purity platform for exosome separation and detection. In this review, we discussed about recent advance in the isolation and detection of exosomes as well as their clinical application with an emphasis on the newly developed techniques for exosome separation and detection. Furthermore, the application of exosomes as potential biomarkers in cancer liquid biopsy was summarized.

## Exosome biogenesis and contents

**Fig. 2:**
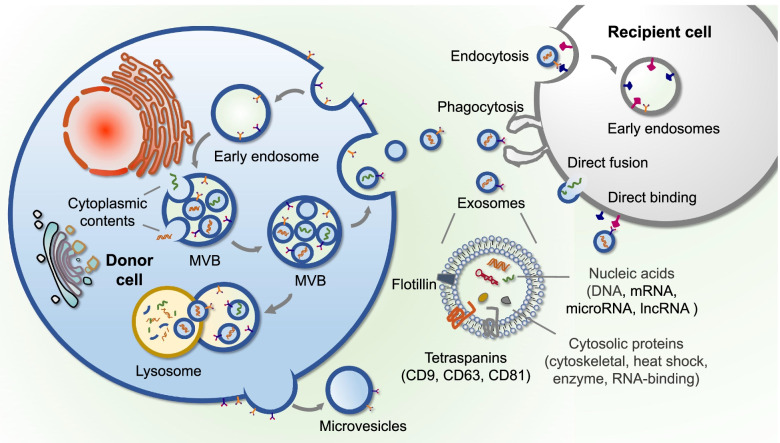
The biogenesis, contents, and internalization of exosomes. Exosomes are vesicles derived from the fusion of multivesicular bodies with plasma membranes. Cytoplasmic contents of donor cells such as nucleic acids and proteins are sorted into exosomes and are delivered to recipient cells through the manner of endocytosis, phagocytosis, direct fusion or direct binding (receptor-ligand interaction)

Exosomes are a heterogeneous group of membrane-structured vesicles actively released by most cells and could be found in many human body fluids, such as blood, saliva, tear and urine [4]. The process of exosome biogenesis involves invagination of plasma membrane, formation of multivesicular bodies (MVBs) and exosome secretion [28]. MVBs are endocytic structures formed by the inward budding of endosomal membranes. Vesicles accumulating inside of MVBs, named intraluminal vesicles (ILVs), are released as exosomes by the fusion of MVBs with plasma membrane [4] (Fig. 2). Extensive studies suggest that donor cell-derived bioactive molecules are enriched in exosomes, which indicates the crucial role of exosomes in genetic information exchange [4, 28]. Notably, the specific RNA components in exosomes have shown great differences compared to those in their parental cells, which may be attributed to the unique process of cargo sorting during exosome formation [29]. The mechanisms of cargo sorting are still unclear, both endosomal sorting complex required for transport (ESCRT)-dependent manner [30, 31] and ESCRT-independent mechanism have been reported to participate in this process [32, 33]. Intriguingly, proteins such as tetraspanins (CD9, CD63 and CD81), heat shock proteins (HSP60, HSP70) and ESCRT-associated components (Alix and TSG101) have been confirmed to be present  in exosomes, which provides certain markers for their identification and detection [34].

Increasing studies have shown that various disease-related proteins and nucleic acids are loaded into exosomes and differentially expressed in tumors of different origin. Hoshino et al. indicated that plasma-derived exosomes could identify specific cancer types and distinguish tumor sources by proteomic analysis. They found that 51 and 19 plasma-derived exosome proteins were specifically identified in pancreatic and lung cancer, respectively [35]. In addition, exosomal CD63 was reported to be high in ovarian cancer while low in lung cancer [36]. CD317 and epidermal growth factor receptors (EGFR) were highly expressed on non-small cell lung cancer (NSCLC)-derived exosomes [37]. Compared with healthy subjects, glypican-1 (GPC-1) was found to significantly increase in serum exosome of patients with pancreatic cancer and could be used for the early detection of pancreatic cancer with 100% diagnostic specificity and sensitivity [38]. In addition to proteins, nucleic acids such as miRNA, mRNA, and lncRNA are differentially distributed in exosomes and could be used as specific cancer biomarkers. For instance, mutant EGFRvIII mRNA was detected in serum exosomes of glioblastoma patients [39]. Zhou et al. reported that the expression level of exosomal miR-15a-5p was 7-19 times higher in endometrial cancer than that in other cancer types [40]. Additionally, high expression of exosomal miR-1247-3p was positively associated with lung metastasis from liver cancer, which indicated a poor outcome [41]. Given that exosomes are secreted by living cells, the specific contents in exosomes could reflect the pathophysiological state of their parental cells, which makes them useful biomarkers for dynamic monitoring of disease progression [42]. In general, cargoes sorted into exosomes can not only supply additional characteristics for their identification, but also provide promising biomarkers for diagnosis, treatment monitoring, and prognosis prediction in patients with cancer, which offers a new tool for liquid biopsy.

## Methods for exosome isolation and enrichment

Due to unique formation manner and specific cargo sorting process, exosomes are heterogeneous in size and molecular contents. Exosome separation and enrichment from complicated biological components is essential for basic study and clinical translation. Up to now, a number of methods have been developed that are significantly varied in the amount and purity of isolated exosomes.

## Conventional isolation methods

### Ultracentrifugation-based separation

As the gold standard for exosome separation, ultracentrifugation is the most commonly used method [43], including differential ultracentrifugation and gradient density ultracentrifugation. Conventional differential ultracentrifugation was first proposed by Johnstone et al. to isolate exosomes from culture medium of reticulocytes [44]. Typically, low-speed centrifugation (300 g) is first employed to remove cellular debris, while 20,000 g speed of centrifugation is utilized to eliminate other large vesicles. A high force (100,000 g) is finally utilized to sediment exosomes. However, this method needs costly instrument and has contamination with aggregated proteins. More centrifugation cycles may obtain a purer outcome but lead to lower recovery. Gradient density ultracentrifugation is a better alternative to obtain exosomes with higher purity [45]. During centrifugation, different sizes of particles from two or more solutions are separated into different layers, whose density increases from top to bottom. Based on this, the method has been applied to separate exosomes, which have been found to float with densities of 1.15 to 1.19 g/mL [45, 46]. Although with higher purity than ultracentrifugation, limitations such as time-consuming process and the requirement of large biofluid volume have largely restrained its use in clinical application.

### Size-based separation

The feature of fixed-range diameters allows for the possibility to separate exosomes by size-based methods. Filtration is one of the size-based approaches for exosome separation by using membrane filters with specific pore sizes, which has the advantages of simple operation and effective purification but disadvantage of low yield [47]. Ultrafiltration is usually applied to concentrate exosomes from large amounts of original materials such as cell culture medium [48]. Currently, the combination of ultrafiltration with ultracentrifugation has been widely employed, in which filtration is used to remove cells and large vesicles while the purification of exosomes is achieved by ultracentrifugation [49]. Moreover, size-exclusion chromatography (SEC) could separate biomolecular components according to the size of sample and pore size of gel. During the separation, large molecules are eluted early, while small molecules or particles directly diffuse into the pores [50]. Anita N Böing et al. developed an SEC-based protocol by cross-linked sepharose CL-2B column, which could efficiently isolate exosomes with a diameter larger than 70 nm from platelet-free supernatant [50]. Guo et al. showed that CL-6B column had better performance than CL-2B column in particle yields and purity of exosomes [51]. Compared with centrifugation and filtration methods, SEC has the advantages of gentle processing and nondestructive outcomes [52]. Moreover, the combination of SEC with ultracentrifugation may have an improved recovery and purity [53].

### Precipitation techniques

As for precipitation techniques, highly hydrophilic polymer is used to competitively bind to water molecules around the exosomal membrane, thereby reducing the solubility and finally achieving low input volume of exosome separation. To date, polyethylene glycol (PEG) is the most commonly used polymer for exosome separation [54]. An exosome purification method called ExtraPEG was proposed by Rider et al., which could rapidly enrich exosomes and obtain sufficient contents harvested from vesicles for downstream analysis [55]. Recently, many commercial kits that rely on precipitation techniques have been developed for exosome isolation and enrichment, such as ExoQuick™ and Total Exosome Isolation. Study from Ding et al. indicated that ExoQuick™ could generate a relatively high yield of exosomes compared with other kits [56]. However, this method has been often criticized for the high cost and contamination of coprecipitated protein aggregates.

## New enrichment methods

**Fig. 3:**
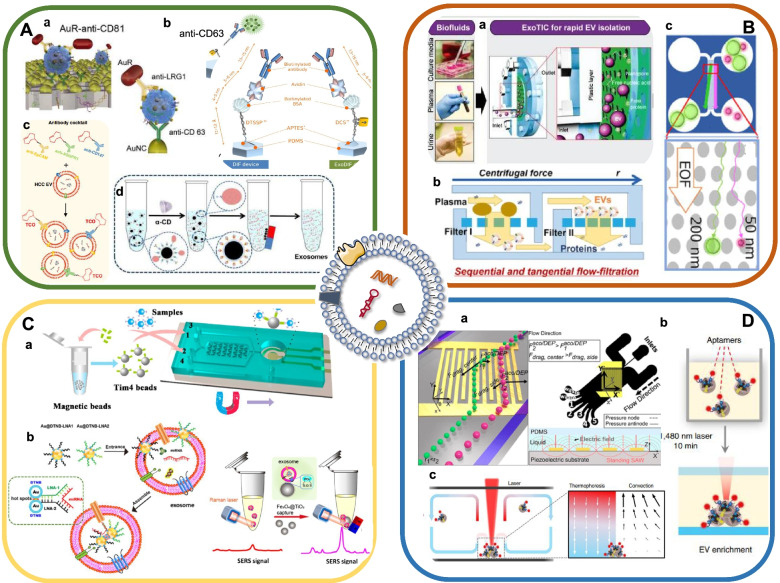
New technologies for the isolation of exosomes. **A** Immunoaffinity/ immunomagnetic enrichment. Copyright 2020 by Yang [58], 2017 by Kang [59], 2018 by Cai [60], 2020 by Sun [61]. **B** Physical feature-based separation. Copyright 2017 by Liu [62], 2019 by Sunkara V [63], 2019 by Hattori [64]. **C** Lipid mediated-separation. Copyright 2018 by Xu [65], 2021 by Jiang [66]. **D** Acoustic-based isolation / Thermophoretic enrichment. Copyright 2021 by Tayebi [67], 2019 by Liu [68], 2021 by Tian [69]

The discoveries of exosome-specific markers and components provide a new avenue for separating exosomes as well as exosome subsets (Fig. 3). Through antibodies and aptamers that specifically target tumor-associated proteins such as GPC-1 and EpCAM (epithelial cell adhesion molecule), exosomes of cancer cell origin could be well distinguished from that of normal cell [57]. Moreover, the applications of microbeads, microfluid chip and thermophoresis enabled the rapid and convenient enrichment of exosomes. Herein, we summarized the advantages and limitations of new approaches for exosome enrichment (Table 1).

**Table 1:** The techniques for exosome separation

Techniques	Methods	Advantages	Disadvantages	Prominent examples	Ref.
Conventional techniques					
Ultracentrifugation-based Separation	Differential ultracentrifugation	High purity; established protocol;	Lengthy process; large sample volume; requires ultracentrifuge	Separation of EVs from reticulocyte culture medium	[44]
	Gradient density ultracentrifugation	High purity;	Lengthy process; large sample volume; requires ultracentrifuge	Sucrose gradient-purified prostasomes	[46]
Size-based Separation	Ultracentrifugation with ultrafiltration	High purity; high yield	Contamination of same-sized vesicles; lack specificity; difficulty in scaling	Separation of urinary exosomes	[49]
	size-exclusion chromatography	High yield; gentle processing	Contamination of same-sized vesicles; lack specificity; difficulty in scaling	Isolation of EVs from platelet-free supernatant of platelet concentrates	[50]
Precipitation	Polyethylene glycol precipitation	Simple; fast isolation	Lack specificity; much contamination; difficulty in scaling	Isolation of exosomes from plasma, cell culture supernatant	[49, 54]
	Commercial kits	Simple; fast isolation	Lack specificity; much contamination; high price	Isolation of exosomes from serum and/or plasma	[56]
Novel techniques					
Immunoaffinity Enrichment	Antibody-conjugated platform	Simple; specificity	High-cost; marker dependent	Enrichment of exosomes from clinical samples	[20, 58, 70]
Magnetic Separation	Antibody-modified magnetic beads	Convenient; high efficiency	High-cost; marker dependent	Separation of exosomes	[36, 71–74]
Physical Feature-based separation	Nanoscale lateral displacement	Reduced membrane blockage; gentle processing	Contamination of same-sized vesicles; lack specificity	On-chip sorting and quantification of exosomes	[75]
	Membrane filter	Gentle processing	Contamination of same-sized vesicles; lack specificity	On-chip isolation of intact extracellular vesicles	[62, 76, 77]
	Deterministic lateral displacement	Continuous accurate and precise separation	Low throughout and the requirement of high voltage	Efficient isolation of extracellular vesicles	[75, 78]
	Size-exclusion chromatography	High yield; gentle processing	Contamination of same-sized vesicles; lack specificity	Efficient isolation of extracellular vesicles	[50, 52, 79]
Lipid Mediated-Separation	Lipid nanoprobe/TiO_2_	Minimal damage	Contamination of other phospholipid membrane vesicles; lack specificity	Efficient isolation of extracellular vesicles	[66, 80]
Acoustic-based microfluidics	Aacoustic radiation force (ARF) and dielectrophoretic (DEP)	Contact-free; high-throughput; continuous separation; wide range of particles	Design and fabrication finer gradations; finer-grade separation of subpopulations	Active sorting of extracellular vesicles	[67, 81]
Thermophoretic Enrichment	Thermophoresis	Free from pre-isolation; simple; fast isolation	Contamination of same-sized vesicles; lack specificity	Efficient isolation of extracellular vesicles	[68, 69]

### Immunoaffinity enrichment

Owing to the priority of simplicity and specificity, immunoaffinity isolation strategy has been used for exosome enrichment in many studies (Fig. 3A). For example, anti-CD81 functionalized microfluidic chip was fabricated by Zhang et al. to isolate exosomes from plasma samples [70]. Yang et al. reported an integrated microfluidic device for exosome separation through forming a sandwich structure of AuNC-exosome-AuR complexes [58] (Fig. 3A_a_). This device achieved a yield of 5 × 10^9^ particles from 5 mL urine sample in 30 min. Nevertheless, the dissociation of captured exosomes remains a big challenge, which arises from the strong affinity between antigen and antibody. To address this challenge, Kang et al. developed an exosome-specific dual-patterned immunofiltration (ExoDIF) device [59]. The biotinylated anti-CD63 antibody was immobilized on the surface of inner channel that sequentially pretreated with 3,3′-dithiobissulfosuccinimidylpropionate (DTSSP), biotinylated BSA, and avidin. As a result, nearly 87.1% of exosomes were captured from high dilution of cell culture media. Notably, the captured exosomes could be dissociated by DTT (dithiothreitol) through breaking the embedded disulfide bond of DTSSP (Fig. 3A_b_).

Recently, EVs on demand chip (EVOD) was proposed by Kang et al., in which the capture of cancer-related exosome subpopulations was achieved by the reaction between tetrazine-conjugated anti-EpCAM/anti-EGFR antibody (TzAb) and TCO (trans-cyclooctene) functionalized microfluidic surface [82]. This chip was able to selectively isolate 76% more EGFR^+^ exosomes from cancer patients than that from healthy donors, which exhibited great potential for the early detection of NSCLC. Sun et al. designed an interesting covalent chemistry-mediated EV click chip to recognize, enrich, and recover hepatocellular carcinoma (HCC)-specific exosomes from plasma samples by multi-marker cocktail (anti-EpCAM, anti-ASGPR1 (asialoglycoprotein receptor 1), anti-CD147) [61] (Fig. 3Ac). The proposed exosome purification system achieved more than 81% of recovery yield and more than 85% of purity, providing a novel liquid biopsy tool to detect hepatocellular carcinoma. Despite great significance, these methods are high-cost and marker-dependent. In addition, more attention should be paid to the non-destructive release of captured exosomes.

### Magnetic separation and enrichment

Magnetic bead-based immunoaffinity enrichment has attracted much attention in recent years due to the advantages of convenience and high efficiency (Fig. 3A). Generally, exosomes are captured by antibody-modified magnetic beads, which are then separated by magnetic force. For example, Fang et al. conducted CD63 antibody-conjugated magnetic nanoparticles to isolate exosomes [71]. Moreover, novel immuno-affinitive superparamagnetic nanoparticles (IS-NPs) were proved to possess high efficiency, which combined anti-CD63 antibodies with superparamagnetic nanoparticles through the interactions between β-cyclodextrin (β-CD) and 4-aminoazobenzene (AAB) [60] (Fig. 3A_d_). α-CD, a competitive agent extracting AAB from the β-CD-AAB inclusion compound, was adapted for the elution of exosomes. As a result, the capture and release efficiency of exosomes from artificial model samples was as high as 80% and 86.5%, respectively. IS-NPs method exhibited higher yield, increased purity and well-retained structural and functional integrity of exosomes than conventional separation methods.

The isolation of exosome subsets, especially cancer-derived exosomes, could be achieved by coupling magnetic beads with antibodies targeting tumor-specific biomarkers. Luo et al. performed a rapid separation and capture of exosomes by immunoaffinity magnetic beads and DNA origami-based aptamer. The quantitative detection of exosomes could be achieved through the combination with DNA fluorescence probe [83]. Li et al. developed a homogenous magneto-fluorescent exosome (hMFEX) nanosensor to separate GPC-1 positive exosomes in 80 μL of plasma from breast cancer patients [84]. He et al. conducted a microfluidic platform to capture tumor-derived exosomes by mixing samples with anti-EpCAM or anti-CA125 antibodies-labeled magnetic beads [36]. In general, the immunomagnetic separation methods hold the potential to facilitate rapid separation and clinical implication of circulating exosomes in desired areas.

### Physical feature-based separation

Size-based microfluidic chip was used to separate exosomes from large cell debris or other membranous vesicles [76, 85] (Fig. 3B). Liu et al. designed a size-based exosome total isolation chip (ExoTIC) in which exosomes ranging in 30-200 nm were enriched and purified by multiple nanoporous membranes [62] (Fig. 3B_a_). Compared with ultracentrifugation and commercial PEG precipitation kits, ExoTIC obtained much higher yields of exosomes from small volumes of human plasma. However, the blockage of membrane pores greatly limited the continuous separation of exosomes. To overcome this problem, Chen et al. introduced an ultrafast-isolation system, EXODUS, which integrated double coupled harmonic oscillations into a dual membrane filter configuration. By periodic negative pressure and air pressure switching, periodic negative pressure oscillations were generated on the nanoporous anodic aluminum oxide membrane, allowing small particles (i.e., proteins and nucleic acids) and fluids to pass through, while larger exosomes remained in the central chamber. Moreover, two pairs of oscillators enabled the resuspension of particles into the liquid via transverse waves and acoustofluidic streaming, which effectively limited fouling and particle aggregation [86]. In addition, tangential flow filtration (TFF) is a technology that effectively reduces the potential of pore clogging due to the perpendicular state of flow direction and filtration direction. Sunkara et al. developed a microfluidic tangential flow filtration device, Exodisc, to separate exosomes from human plasma and urine, which showed better exosome yield compared to conventional methods [63] (Fig. 3B_b_). Deterministic lateral displacement (DLD) has been used for exosome separation since particles with different sizes perform distinct trajectories in a platform with certain angle-displayed micropillars. Wunsch et al. proposed a nanoscale lateral displacement (nano-DLD) array, in which larger vesicles laterally displaced across the array and were collected at a side channel while smaller vesicles flew out of the array in a zigzag mode, finally achieving the collection of urine-derived exosomes [75]. However, DLD was limited by low throughout and the requirement of high voltage and the density and stiffness of vesicles may interfere with DLD-based exosome isolation.

### Lipid-based separation

Lipid molecules on the surface of vesicles enable affinition-mediated exosome capture (Fig. 3C). Wan et al. reported a lipid nanoprobe for the rapid separation of exosomes from plasma [80]. The lipid bilayer of exosome was labeled with biotin-tagged 1,2-distearoyl-sn-glycero-3-phosphethanolamine-poly (ethylene glycol) (DSPE-PEG) probes in which DSPE was inserted into the exosome membrane by hydrophobic effect and PEG provided solubility in the aqueous phase. NeutrAvidin-coated magnetic sub-micrometer particles were used for the collection of exosomes through avidin-biotin affinity. As a result, it took only 15 min to isolate exosomes, which greatly shortened the isolation procedure [87]. Notably, cholesterol-PEG_1000_ of ~ 6.4 nm was considered to minimize steric hindrance in surface immobilization of lipid nanoprobes and thus achieved a much higher capture efficiency. Additionally, molecules absorbed to lipids on exosome membrane have also been applied to capture exosomes [88]. T-cell membrane protein 4 (Tim-4), which has high affinity to phosphatidylserine (PS), has been verified to facilitate simple exosome separation from serum samples, making it easier for rapid downstream analysis [65, 89] (Fig. 3C_a_). In addition, the affinity interaction of TiO_2_ shell and phosphate groups of exosomes is a novel strategy for the enrichment of phosphorylated peptide. Pang et al. used Fe_3_O_4_@TiO_2_ nanoparticles to enrich and separate exosomes within 5 min with a capture efficiency of 96.5% [90]. The combination of Fe_3_O_4_@TiO_2_ nanoparticles and surface-enhanced Raman scattering (SERS) tags could enrich exosomes and achieve in-situ qualification of target miRNAs simultaneously [66] (Fig. 3C_b_).

### Acoustic-based isolation method

Acoustic-based microfluidics is a simple and efficient method for exosome separation. Typically, ultrasonic waves are applied to samples and the particles undergo different forces and are separated depending on their physical properties such as size and density [81] (Fig. 3D). Anson et al. utilized the ultrasonic waves scattering between micrometer-sized seeding particles and nanoparticles in a resonant cavity to enrich exosomes. The integrated acoustic device enabled fast operation, non-contact and continuous separation of exosomes from urine and plasma samples [91]. Wu et al. developed an acoustofluidic platform for direct, label-free, and contact-free enrichment of exosomes from whole blood. The device integrated two separation modules, one of which was able to remove particles larger than 1 μm in diameter and the other one could isolate exosomes from larger microvesicles and other particles [92]. Recently, Gu et al. reported an acoustically driven spinning droplets device, in which slanted interdigitated transducers (IDTs) were used to allow nanoparticles to move according to the sound waves of varying frequencies. Particles of fixed size could be specifically concentrated by placing two droplets of different sizes next to each other. As a result, the method could isolate exosomes from 5 μL samples in less than 1 min [93]. In addition, Tayebi et al. combined acoustic radiation force (ARF) with dielectrophoretic (DEP), in which high frequency (> 10 MHz) interdigital transducer was placed in the flow path to simultaneously generate ARF and dielectrophoretic force field. Particles in the medium presented lateral translation by the competition between fluid drag forces, ARF, and DEP force fields, resulting in an active separation of extracellular vesicles [67] (Fig. 3D_a_).

### Thermophoretic enrichment

Thermophoresis refers to a phenomenon that particles migrate from space with high- temperature to low-temperature areas via a temperature gradient induced by localized laser heating. To address the challenge of time-consuming isolation and purification procedures, Liu et al. developed a sensitive thermophoretic method to enrich tumor-derived exosomes. By using aptamers that targeted tumor-specific markers, thermophoresis could achieve rapid isolation and enrichment of exosomes from other components without exosome pre-isolation (Fig. 3D). The accumulation of exosomes produced an amplified fluorescence signal of aptamers, which enabled profiling of surface biomarkers of exosomes as well as detection of miRNAs [68] (Fig. 3D_b_). Tian et al. performed a similar assay to analyze exosomes in 1 μL plasma, which offered a low-cost, sensitive method for liquid biopsy in metastatic breast cancer [69] (Fig. 3D_c_).

## Exosome characterization

As suggested in the Minimal information for studies of extracellular vesicles 2018 (MISEV2018), the identification of exosomes should include western blot verification of exosome-specific markers and at least two methods for characterization of single exosome [94, 95].

### Visible characterization

**Fig. 4:**
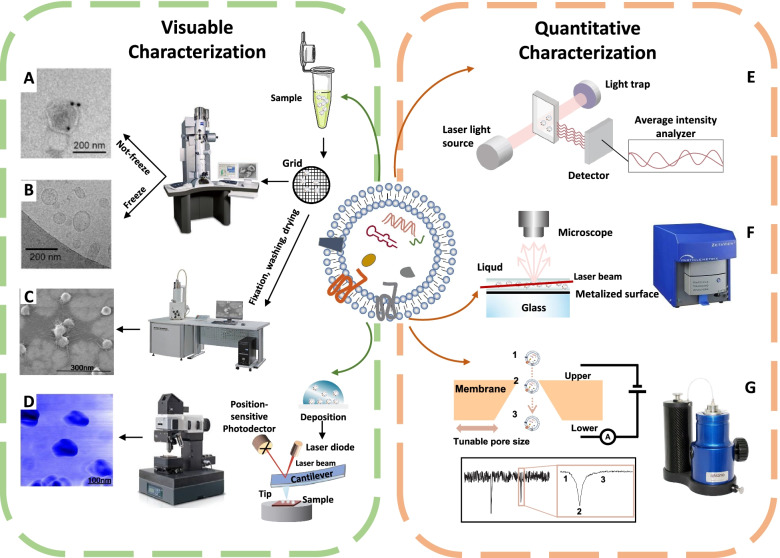
Technologies for the characterization of exosomes. **A** Transmission electron microscopy. Copyright 2020 by Li [99]. **B** Cryo-electron microscopy. Copyright 2018 by Tian [97]. **C** Scanning electron microscopy. Copyright 2010 by Sharma [96]. **D** Atomic force microscopy. Copyright 2010 by Sharma [96]. **E** Dynamic light scattering. **F** Nanoparticle Tracking Analysis. **G** Tunable resistive pulse sensing

Transmission electron microscopy (TEM) is considered as the common method to identify and characterize a single exosome with typically cup-shaped structure (Fig. 4A) [62]. For scanning electron microscopy (SEM), images are presented by collecting the electrons ejected from the samples (Fig. 4C) [96]. Notably, the morphology of exosomes may be affected by dehydration during sample handling procedures. On the contrary, cryo-electron microscopy (cryo-EM) is a better choice because it avoids sample fixation and dehydration. Exosomes are analyzed at a very low temperature and exhibit a round structure that is different from TEM images [97] (Fig. 4B). In addition, atomic force microscopy (AFM) could provide information on both surface morphology and material properties (stiffness, adhesion) by amplitude modulation and phase modulation [96] (Fig. 4D). Moreover, specific exosome membrane markers functionalized AFM tips permit the identification and detection of proteins in single exosome [96, 98].

### Quantitative characterization

Dynamic light scattering (DLS) is a technique for measuring the distribution of exosome size and zeta potential. The changes of scattered light interfere and intensity can be identified by a sensor, allowing for estimating the size distribution of particles [100–102] (Fig. 4E). However, the contamination of protein aggregates and large vesicles makes it challenging for DLS to distinguish them from exosomes [103]. Nanoparticle tracking analysis (NTA) is a widely used method for determining the concentration and size distribution of particles (Fig. 4F). Particles are illuminated by a beam of light and the scattering light signals are collected by optical microscope [100, 104]. Tunable resistive pulse sensing (TRPS) detects the electrical signals generated by changes in ion conductivity when exosomes pass through pores filled with conductive media [105] (Fig. 4G).

## Techniques for detecting exosome contents

### Conventional protein analysis

Both membrane and cytoplasmic proteins could be detected in exosomes [4]. Some membrane proteins on exosomes are involved in cancer development and progression and thus have been used as targets for exosome isolation and purification [28, 106]. Western blot and enzyme-linked immunosorbent assay (ELISA) are two regular approaches widely used to detect exosomal proteins [7, 107]. However, these methods are faced with complex procedure and low sensitivity problems [108].

## New protein detection methods

### Colorimetric detection

**Fig. 5:**
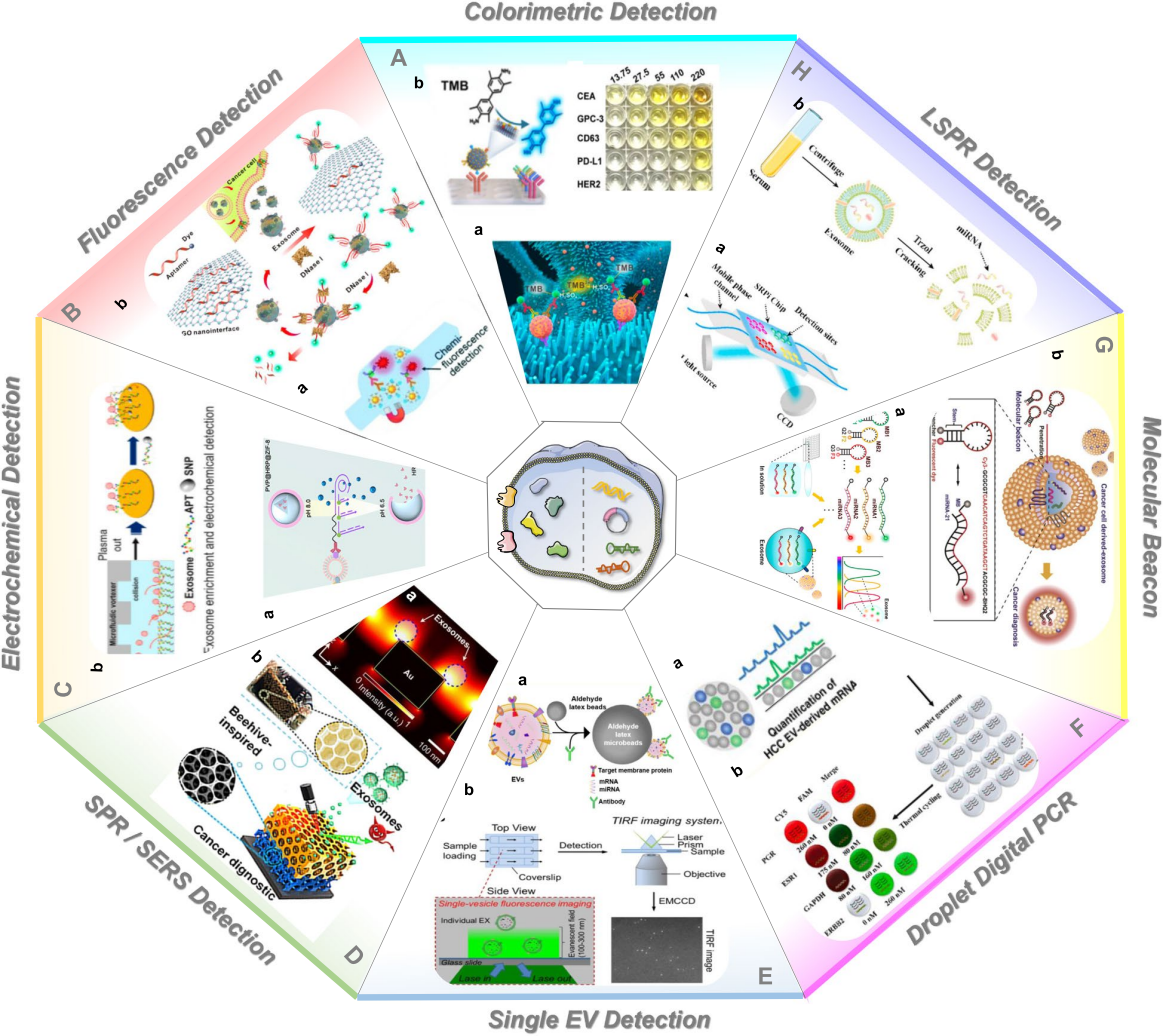
New methods to detect the contents of exosomes. **A** Colorimetric detection. Copyright 2018 by Chen [110], 2020 by Di [112]. **B** Fluorescent detection. Copyright 2014 by He [36], 2018 by Jin [113]. **C** Electrochemical detection. Copyright 2020 by Cao [114]. 2020 by Kashefi-Kheyrabadi L [115]. **D** SPR/SERS detection. Copyright 2014 by Hyungsoon Im [116], 2020 by Dong [117]. **E** Single exosome detection. Copyright 2019 by Wang [118], 2019 by He [119]. **F** ddPCR. Copyright 2020 by Sun [61], 2021 by Liu [120]. **G** Molecular beacon., Copyright 2016 by Ji HyeLee [121], 2015 by Ji HyeLee [122]. **H** LSPR detection. Copyright 2021 by Wu [123]

Up to now, multiply novel techniques have been developed to detect exosomal proteins (Table 2). Colorimetric detection is a method to determine the content of a component by comparing or measuring the color intensity of chromogenic substances (Fig. 5A). Similar to other nanomaterials like Fe_3_O_4_ NPs and AuNPs, DNA has the ability of significantly increasing the peroxidase activity of single-walled carbon nanotubes (s-SWCNTs). Xia et al. developed a visible and simple method for the detection of exosomes [109]. Briefly, CD63 aptamer improved the minic peroxidase activity of s-SWCNTs and effectively catalyzed oxidation of 3,3′,5,5′-tetramethylbenzidine (TMB), resulting in a colorless solution turning blue. On the contrary, the addition of exosomes induced the release of aptamer from the surface of nanotubes and the color of solution to turn from dark to light, which could be observed by naked eye or UV-visible spectrometry with a detection limit (LOD) of 5.2 × 10^5^ particles/μL. As previously discussed, ZnO-chip was designed to effectively isolate exosomes. After incubation with primary antibody mixture (anti-CD9/CD63 antibody), exosomes were recognized by HRP (horseradish peroxidase)-labeled secondary antibody (Fig. 5A_a_). Finally, a minimal detectable concentration of 2.2 × 10^4^ particles/μL was obtained by UV-visible spectrometry or microplate analyzer [110]. Liang et al. designed a microfluidic system integrated with double membrane filter and ELISA to detect the content of exosomes in urine samples of bladder cancer patients [111]. In addition to common exosomal proteins, colorimetry could be applied to detect cancer-specific proteins as well. For instance, a PSA (prostate-specific antigen) aptamer-based sensor was used for the visual detection of prostate cancer-specific exosomes in 500 μL human plasma [72]. Moreover, Di et al. reported a nanozyme-assisted immunosorbent assay (NAISA), which enabled sensitive and rapid multiplex profiling of exosomal proteins [112] (Fig. 5A_b_). The surface proteins of exosomes could be specifically captured by antibodies immobilized on the surface of a microplate and catalyzed a colorimetric reaction. Signal intensity obtained from microplate reader is proportional to the number of target proteins. As a result, NAISA allowed the rapid profiling of multiple exosomal proteins such as CD63, CEA (carcinoembryonic antigen), GPC-3 (Glypican-3), PD-L1 (programmed death-ligand 1), and HER2 (human epidermal growth factor receptor 2) from clinical samples.

**Table 2:** New technologies for exosomal protein detection

Methods	Exosome sources	Sample volume	Sensing mechanism	Sensing substances	Detection limit	Ref.
Colorimetric Detection	MCF-7 cells and breast cancer patient’s serum		H_2_O_2_-mediated oxidation of TMB	s-SWCNTs; CD63-specific aptamer	5.2 × 10^5^ particles/μL	[109]
	Cell-culture medium and prostate cancer patient’s plasma	500 μL	H_2_O_2_-mediated oxidation of TMB	Aptamer-capped Fe_3_O_4_ nanoparticles	3.58 × 10^6^ particles/mL	[72]
	Urine	100 mL	H_2_O_2_-mediated oxidation of TMB	Biotinylated anti-CD63 antibody; streptavidin-labeled HRP	35.0 AU/mL	[76]
	MCF-7 cells and cancer patient’s serum	100 μL	H_2_O_2_-mediated oxidation of TMB	CD9, CD63 antibody mixture; HRP-labeled secondary antibody	2.2 × 10^4^ particles/μL	[110]
	BeWo cell		H_2_O_2_-mediated oxidation of TMB	Au-NP; Fe_2_O_3_NC	10^3^ exosomes/mL	[148]
Fluorescence Detection	Plasma specimens from NSCLC and OVCA patients	30 μL	Chemifluorescence reagents	EpCAM, IGF-1R or CA125 antibodies; AP-conjugated secondary antibody, and the DiFMUP substrate	0.281 pg/mL; 0.383 pg/mL	[36]
	SKOV3 cells and plasma of OVCA patients	10 μL	The reaction of SβG with FDG	Biotin conjugated detection antibodies and streptavidin conjugated SβG	21 exosomes/μL	[149]
	MCF-7 and MDA-MB-231 cell culture medium	1 mL	Fluorescent carbocyanine dye (DiO)	CD63 antibody functionalized microbead and DIO labelling		[150]
	Cell culture supernatant and serum from pancreatic cancer patients		Fluorescent carbocyanine dye (DiO)	CD63 antibody-functionalized EXOchip		[20]
	MCF-7 cells and blood samples from cancer patients	100-300 μL	Fluorescent second antibody	Anti-EpCAM antibody and Alexafluor®647-conjugated secondary antibody		[59]
	Cell-culture medium and plasma from breast cancer patients		Fluorescent second antibody	CD63 antibody-coated magnetic beads; fluorescent dye-conjugated antibodies	10^7^ particles/μL	[151]
	A549 cancer cell line and plasma samples of lung cancer patients	0.5 μL	Fluorescent aptamer	TMR-aptamer	500 particles/μL	[152]
	Serum samples		Fluorescent aptamer	CD63 aptamer-modified magnetic beads; Cy3-labeled short sequence	1.0 × 10^5^ particles/μL	[126]
	Cancer cell line and plasma samples	500 μL	Fluorescent aptamer	TPE-TAs/aptamer complexes; graphene oxide surface	3.43 × 10^5^ particles/μL	[99]
	MDA-MB-231 cell-culture medium and plasma from breast cancer patients	80 μL	Fluorescence quenching	GPC-1 antibody coated magnetic beads; CD63 aptamer	6.56 × 10^4^ particles/μL	[84]
	Cancer cell line and serum samples		Fluorescence quenching	FAM-labeled aptamers; graphene oxide	1.6 × 10^5^ particles/mL	[113]
	Cancer cell line and blood samples		Fluorescence quenching	Anti-CD63-PE/MoS2–MWCNT	14.8 × 10^5^ particles/mL	[153]
Electrochemical Detection	Ovarian cancer cell lines and plasma from patients with ovarian cancer	10 μL	Integrated magneto-electrochemical sensor	Immunomagnetic beads; HRP-labeled secondary antibody	3 × 10^4^	[128]
	Plasma samples	20 μL	Electrochemical biosensor	Immunomagnetic beads; probing antibodies		[129]
	Cell-culture medium and blood samples from breast cancer patients		Electrochemical biosensor	Anti-PD-L1-linked DNA strand; PVP@HRP@ZIF-8	334 particles/mL	[114]
	HepG2 cells and human serum of liver cancer patients	30 μL	Aptamer-based biosensors	CD63 aptamer and mimicking DNAzyme sequence	4.39 × 10^3^ particles/mL	[65]
	Culture medium of HepG2 cells		Aptamer-based biosensors	NTH-assisted aptasensor	2.09 × 10^4^/mL	[154]
	Cell-culture medium and serum		Aptamer-based biosensors	Aptamer-magnetic bead bioconjugates; electroactive Ru (NH3)63^+^	70 particles/μL	[155]
	Cellular supernatant and human plasma samples		Aptamer-based biosensors	anti-CD63 antibody modified gold electrode and a gastric cancer exosome specific aptamer	9.54 × 10^2^/mL	[156]
	Human hepatoma cell lines MHCC97H/L and mouse melanoma cell lines B16-F1/10		Antibody microarray SPRi sensor	Anti-CD9, CD41b,21 and tyrosine kinase receptor MET8a antibodies immobilized gold-coated glass sensor chip		[157]
SPR Detection	MCF-7 breast cancer cells and MCF-10A normal breast cells		SPR-based aptasensor	Dual gold nanoparticle-assisted signal amplification	5 × 10^3^ exosomes/mL	[158]
	Human NSCLC cell lines, normal lung cell and plasma	1.5 mL	Bioaffinity interactions of antibodies and different recognition sites	Antibodies modified-gold chip and different recognition sites	10^4^ particles/μL	[159]
	Urine samples from lung cancer patients and controls	500 μL	SPR-induced improved scattering intensity	Anti CD81/LRG1 antibody modified nanoporous gold nanocluster membrane; second antibody-conjugated Au nanorod probes	< 1000 particles/mL	[58]
	Breast cancer cell line and serum	250 μL	SPR-induced improved scattering intensity	Anti-HER2-functionalised SPR chip	8280 exosomes/μL	[160]
	Cancer cells and serum and the CSF of an orthotopic mouse model		Strong localization of surface plasmon polaritons	TIC-AFM and TiN–NH-LSPR biosensors	5.29 × 10^−1^ μg/ml; 3.46 × 10 ^-3^ μg /ml	[161]
	Breast cancer cells and normal breast cells; plasma from HER2-positive breast cancer patients		Raman reporters	Gold-coated glass microscopy slide; QSY21-coated gold nanorods	2 × 10^6^/mL	[134]
SERS	Plasma of cancer patients	400 μL	P-O bond signature	Beehives-like Au-coated TiO_2_ macroporous inverse opal		[117]
	Cell-culture medium and serum sample	4 μL	MBA signature	Fe_3_O_4_@TiO_2_ nanoparticles; anti-PD-L1 antibody modified Au@Ag@MBA	1 PD-L1 exosome/μL	[90]
	Normal and lung cancer cell lines; plasma		Deep learning	Deep learning model		[162]
	Cell-culture medium; serum and plasma	< 1 μL	Enrichment of aptamer-bound EVs	Seven aptamers targeting specific proteins; machine-learning algorithm	3.3 × 10^3^/μl	[68]
CRISPR/Cas-assisted detection	A549 cell-culture medium and serum from lung cancer patients		CRISPR/Cas12a	CD63 aptamer; CRISPR/Cas12a	Linear range of 3 × 10^3^–6 × 10^7^ particles/μL	[136]
	SUNE2 cell-culture medium and serum from NPC patients	50 μL	CRISPR/Cas12a	Nucleolin and PD-L1 aptamers; CRISPR/Cas12a	10^2^ particles/μL	[137]
	SUNE2 cell-culture medium and serum from NPC patients		CRISPR/Cas12a	CD109 and EGFR aptamers; CRISPR/Cas12a	10^2^ particles/μL	[138]
Single EV Analysis	Human serum	10 μL	Rolling circle amplification	ssDNA-assisted single EV detection platform	82 vesicles/μL	[139]
	T3M4 pancreas cancer line and serum from PDAC patients	10 mL	Flow cytometry	Aldehyde/sulfate latex beads; anti-GPC-1 antibody and Alexa-488-tagged antibody		[38]
	Breast cancer cell lines and serum of breast cancer patients	500 μL	Flow cytometry	Aldehyde/sulfate latex beads; anti-EpCAM or anti-HER2 antibody; Alexa-488- or − 594-tagged secondary antibodies		[141]
	Human cell lines and serum of glioma patients	250 μL	Flow cytometry	Aldehyde/sulfate latex beads; anti-EGFR or anti-CXCR4 antibody		[118]
	HCT15 cell-culture medium and plasma	500 μL	Nano-flow cytometry			[142]

*OVCA* Ovarian cancer, *PGR* Progesterone receptor, *ESR1* Estrogen receptor 1, *ERBB2* erb-b2 receptor tyrosine kinase 2

### Fluorescence detection

Fluorescence spectrophotometry is a method for substance identification and content determination according to the positivity and intensity of fluorescence spectral line (Fig. 5B). He et al. conducted a microfluidic chip to detect surface and intravesicular biomarkers from 30 μL plasma samples. This chip obtained a markedly improved detection sensitivity of IGF-1R (type 1 insulin growth factor receptor), 0.281 pg/mL of IGF-1R and 0.383 pg/mL of p-IGF-1R, which was 100-fold higher than that achieved by ELISA [36] (Fig. 5B_a_). Liu et al. developed an immunosorbent assay, in which immunomagnetic beads were utilized to capture exosomes, followed by the conjugation of an enzymatic reporter, which could produce a fluorescent signal for quantitation of GPC-1^+^ exosomes in the droplet microfluidic system [124]. Wei et al. proposed similar single molecule array (SiMoa) platform, by which universal exosomes and tumor-derived exosomes could be ultrasensitively detected with an LOD of 34 particles/μL and 25 particles/μL, respectively [125]. Yu et al. conducted a CD63 aptamer-based detection method. CD63 on exosomes could bind to aptamers modified on the surface of magnetic beads, resulting in the shedding of a Cy3-labeled short sequence into the supernatant. The fluorescence intensity in the supernatant was used to quantify exosomes in complex clinical samples [126].

Graphene oxide has the ability of quenching fluorescence via fluorescence resonance energy transfer (FRET) when conjugated with fluorescent dyes. For instance, the fluorescence of FAM-labeled aptamers was quenched when absorbed onto graphene oxide membranes, while target exosomes competitively bound to aptamers and re-exhibited fluorescent signals with an LOD of 1.6 × 10^5^ particles/mL [113] (Fig. 5B_b_). Li et al. developed a facile fluorescent aptasensor based on aggregation-induced emission luminogens (AIEgens). Graphene oxide absorbed (tetrafrylene-containing tertiary amine) TPE-TAS/aptamer complex allowed fluorescence quenching in the absence of exosomes. When target exosomes were introduced, the aptamer preferentially bound to its target, resulting in the separation of the TPE-TAS/aptamer complex from the graphene oxide surface, followed by a “turn-on” fluorescence signal. The linear range of tumor-derived exosomes was around 4.0 × 10^5^-1.8 × 10^7^ particles/μL under optimized conditions [99].

### Electrochemical detection

Electrochemical detection is used to detect the analytes by measuring the electrochemical potential or current of the sample, which has the advantages of high sensitivity and wide measurement range (Fig. 5C). In recent years, electrochemical biosensors have been developed by researchers since the altered electrochemical signals could quantify exosomes when recognition elements such as antibody and aptamer specifically bind to exosomes [127, 128]. Cao et al. proposed an electrochemical biosensor to accurately detect PD-L1^+^ exosomes [114] (Fig. 5C_a_). Exosomes were firstly captured by anti-CD63 functionalized magnetic beads and then bound with anti-PD-L1-linked DNA strand, introducing a hyperbranched rolling circle amplification (HRCA). The HRCA could decrease the environmental pH, leading to the decomposition of PVP@HRP@ZIF-8 and release of HRP, which resulted in amplified electrochemical responses and thus achieved the identification and detection of cancer-derived PD-L1^+^ exosomes. This biosensor displayed a wide dynamic range for PD-L1^+^ exosomes (1 × 10^3^ to 1 × 10^10^ particles/mL) and the detection limit was 334 particles/mL. Jeongmin et al. reported a HiMEX approach which integrated magnetic exosome separation and electrochemical detection of exosome-bound proteins after enzymatic amplification. The combined detection of tumor biomarkers (EGFR, EpCAM, CD24 and GPA33) in exosomes from 20 μL plasma samples were helpful for the diagnosis and monitoring of colorectal cancer [129]. Electrochemical methods, especially aptamer-based biosensors have shown great potential in the detection and profiling of exosomal proteins [65]. Kashefi-Kheyrabadi et al. introduced a detachable microfluidic device implemented with an aptamer-based electrochemical biosensing method (DeMEA). In this system, aptamer targeting EpCAM was immobilized on the electrode surface that was pre-electroplated with gold nanostructures and microfluidic vortexes could increase the collision between exosomes and sensing surface (Fig. 5C_b_). Consequently, DeMEA was able to quantify exosomes from plasma samples of breast cancer patients at different stages, which provides a highly sensitive and early detection of cancer-specific exosomes [115].

### Surface plasmon resonance detection

Surface plasmon resonance (SPR) is a physically optical phenomenon caused by total reflection of light at the metal film/liquid level interface to analyze molecular interactions (Fig. 5D) [130, 131]. Im et al. developed a nano-plasmonic exosome (nPLEX) assay based on transmission SPR through periodic nanohole arrays (Fig. 5D_a_). With functionalized antibodies in each array, the nPLEX sensor displayed spectral shifts or intensity changes that were proportional to the levels of target exosomal proteins [116]. In another study, the markedly improved scattering wavelength shift and scattering intensity were observed on AuNC-Exosome-AuR due to the plasmon effect [58]. The differential expression of LRG1 (leucine rich alpha-2-glycoprotein 1) in urinary exosomes between lung cancer patients and healthy individuals was evaluated by anti-LRG1 antibody-conjugated AuR probes. Another surface plasmon resonance imaging (SPRi)-based biosensing assay was developed by Fan et al. The bioaffinity interactions between antibodies (anti-CD63/anti-EGFR/anti-EpCAM) modified-gold chip and different recognition sites permitted the multiplex characterization of NSCLC-derived exosomes. The LOD of this biosensor was estimated to be 10^4^ particles/μL. Despite multiple merits, the broad applications of nanoplasmonic biosensors are restrained by the difficult fabrication of nanostructures. To address this challenge, Liu et al. developed an intensity-modulated SPR biosensor free from nanostructure. In this sensor, the reflection intensity and reference intensity of lasers were recorded by two photodetectors and were used to quantify the expression levels of exosomal proteins, which exhibited a higher detection sensitivity than ELISA [132].

### Surface enhanced Raman scattering

Surface enhanced Raman scattering (SERS) is capable of enhancing the Raman signal of small molecules attached to the rough metal surface through electromagnetic and chemical mechanisms [133] (Fig. 5D). A new assay was reported for the real-time detection and protein profiling of exosomes. Generally, gold-plated slides combined with 3D-printed antibody arrays were fabricated to capture exosomes, and QSY21-coated gold nanorods were used as the label agent to quantitatively detect target proteins. The levels of plasma-derived exosomes of breast cancer patients were quantitatively determined by using this assay targeting HER2 and EpCAM. The proposed 3D-printed array template enabled cheap, portable, and easily available establishment of detection platform, providing a new strategy for the development of novel cancer liquid biopsy [134]. Dong et al. showed that the analysis of protein phosphorylation status may provide new possibilities for cancer diagnostics [117] (Fig. 5D_b_). A beehives-like Au-coated TiO_2_ macroporous inverse opal structure was developed to capture and analyze exosomes without any labeling process. The intensity of 1087 cm^− 1^ SERS peak referred to the P-O bond within the phosphoproteins of exosomes and the intensity of peak was at least two times from plasma of cancer patients than that from healthy donors. However, the above-mentioned two assays both require the pre-separation of exosomes, which greatly hinders the rapid analysis. Pang et al. presented a simple immunoassay to capture and analyze exosomal PD-L1 directly from serum samples [90]. Fe_3_O_4_@TiO_2_ nanoparticles were designed to isolate exosomes and anti-PD-L1 antibody modified Au@Ag@MBA SERS tags were applied for exosomal PD-L1 labeling and SERS detection. This assay was confirmed to quantify exosomal PD-L1 in 4 μL serum sample within 40 min.

### CRISPR/Cas system-assisted detection

In CRISPR/Cas9 system, the Cas9 nuclease could efficiently shear the double-stranded DNA (dsDNA) sequence by recognizing specific complementary dsDNA containing protospacer adjacent motif (PAM) sequences with the help of guide RNA (gRNA) [135]. Through aptamers that specifically target exosomal proteins, the detection of proteins could be transformed into the quantification of nucleic acids. Recently, Zhao et al. reported a detection method which combined aptamer-based exosomal membrane protein recognition with CRISPR/Cas12-assisted fluorescence signal amplification [136]. In this system, CD63 aptamer specifically targeted exosome membrane proteins, triggering the conformational change of aptamer and release of blocker strands (with complementary sequences to aptamer). The released blocker was then recognized by CRISPR/Cas12a, resulting in the trans-cleavage toward TaqMan probe and the separation of fluorescence reporter group and quenching group, finally leading to the generation of amplified fluorescence signal. As a result, this method achieved a linear detection range of 10^3^-10^7^ particles/μL and was successfully applied in the direct detection of plasma exosomes without ultracentrifugation. Xing et al. developed an apta-HCR-CRISPR assay to detect circulating nucleolin^+^ or PD-L1^+^ exosomes from 50 μL serum of nasopharyngeal carcinoma cancer (NPC) patients [137]. Nucleolin or PD-L1-targeted aptamer was first amplified by HCR (hybridization chain reaction) to produce a long-repeated CRISPR-targetable DNA unit. Through collecting the fluorescence signal induced by collateral cleavage activities of CRISPR-Cas12a, the assay enabled a detection limit of 10^2^ particles/μL. A similar assay was conducted by Li et al. for the ultrasensitive detection of CD109^+^ and EGFR^+^ exosomes [138]. CRISPR/Cas system is expected to be a sensitive tool for the identification and quantification of exosomal proteins and may contribute to the diagnosis and therapeutic monitoring of cancer.

### Single exosome detection

Intrinsic heterogeneity is one of the main factors hindering exosome analysis in body fluids. Single exosome detection may provide more accurate information of tumor progression (Fig. 5E). Guo et al. presented an ssDNA-assisted single exosome detection platform. Rolling circle amplification (RCA) contributed to an amplified fluorescence signal from the surface protein, enabling easy visualization of individual exosomes with an LOD of 82 vesicles/μL [139]. In addition, Liu et al. developed a λ-DNA and aptamer-mediated approach, allowing for two-dimensional analysis of single exosome by size and tumor-associated marker expression [140].

Since nanosized exosomes cannot be sensitively identified by conventional flow cytometry, aldehyde/sulfate latex beads have been used to bind to vesicles, which are then stained with fluorescent antibodies and characterized for their protein markers. For example, anti-GPC-1 antibody and Alexa-488-tagged secondary antibody were introduced to the exosome-attached beads and the percentage of positive beads was therein referred as the percent of GPC-1^+^ exosomes [38]. In addition, the detection of expressed EpCAM, HER2 and EGFR in exosomes indicated their potential role in cancer diagnostics [118, 141] (Fig. 5E_a_). Moreover, the development of nano-flow cytometry has provided a new option for multi-parameter analysis of single particle. The expression of CD9, CD63, CD81, CD235a, CD45, CD41a and CD144 of single exosome was measured via immunofluorescent labelling using nano-flow cytometry to evaluate the quality of exosome preparations isolated by six different methods. Liu et al. used nano-flow cytometry to analyze the expression of CD9, CD63, CD81, CD47, CD45, CD24, and EpCAM in tear-derived exosomes and found that the exosome concentration in tear fluid was approximately 100-fold higher than that of plasma exosomes [142].

## Conventional nucleic acids analysis

In addition to protein cargoes, nucleic acids are encapsulated in exosomes as well. RNAs represent the major nucleic acid cargo of exosomes, which have shown the potential to be specific biomarkers for cancer diagnosis and prognosis prediction [2, 143, 144]. Nevertheless, the accuracy and feasibility of detecting exosomal nucleic acids are often hampered by their low abundance. To quantify the expression levels of exosomal nucleic acids, techniques such as qRT-PCR (real-time quantitative reverse transcription PCR), microarray, and next-generation sequencing (NGS) have been used. Despite high sensitivity, qRT-PCR can only be used to detect nucleic acids with known sequences [145]. NGS is beneficial for high-throughput discovery and quantitation of unknown exosomal RNA transcripts. However, shortcomings such as high cost, huge amount of data and complexity of building libraries need to be addressed [146]. Through the complementary combination of hybridized probes and target genes, microarrays can analyze thousands of nucleic acids in exosomes at one time but have the disadvantages of low sensitivity [147]. To overcome these limitations, more efforts are being devoted to developing highly sensitive and convenient methods for exosomal nucleic acid detection (Table 3).

**Table 3:** New technologies for exosomal nucleic acid detection

Methods	Exosome sources	Sample volume	Nucleic acids	Detection mechanism	Advantages	Disadvantages	Ref.
Droplet digital PCR	Urine	2 mL	miRNA; gene variation	Nucleic acid amplification of droplets in an oil emulsion	Absolute quantification; small sample volume; high accuracy and sensitivity;	High-cost; limited throughout; complex operation	[163]
	Cerebrospinal fluid of GBM patients	1 mL	*IDH1* mutation				[164]
	Plasma samples of HCC patients and control cohorts	90 μL	HCC-specific mRNA				[61]
	Cancer cell lines and patient plasma	2 μL	*GAPDH* mRNA				[165]
	Human plasma		miR-15a-5p				[40]
	Human plasma		PGR mRNA; ESR1 mRNA; ERBB2 mRNA				[120]
	Clinical blood	1.5 mL	EV-lncRNA of SLC9A3-AS1 and PCAT6				[166]
	Serum	100 μL	circHIPK3 and circSM ARCA5				[167]
Molecular beacons	Cancer cells and human serums	35 μL	miRNA-21	Fluorescent, enzyme-labeled oligonucleotide probes identifying and detecting nucleic acid with complementary sequences	High specificity, simplicity; low background fluorescence; rapid detection	High-cost; limited throughout	[122]
	Breast cancer cell line and human plasma		miR-21; miR-375; and miR-27a				[121]
	Prostate cancer cells and human urine		miRNA-375 and miRNA-574-3p				[168]
	Human plasma	10 μL	miR-1246				[169]
	RBC-derived EVs		miRNA-451a				[170]
	PCA cell		miR-21				[151]
DNA tetrahedron probe	Serum		miR-21	Leverage localized reaction and cascade amplification	High specificity and sensitivity	High-cost	[171]
	Plasma	1 mL	miR-1246; miR-221; miR-375; miR-21				[172]
SPR Detection	Pancreatic cancer cells and plasma	50 μL	miR-10b	The change of dielectric constant caused by molecule adsorption on the heavy metal film	High specificity and sensitivity; label-free	Nonspecific adsorption	[173]
	Plasma		miRNA				[123]
	Mouse serum		miR-10b				[174]
Single Vesicle Analysis	Serum		hsa-miRNA-21	Single-vesicle imaging	Direct visualization; acknowledgement of heterogeneity at the single-vesicle level	Nonspecific adsorption	[119]
Thermophoretic Detection	Serum	0.5 μL	miRNA	Nanoflare induced amplified fluorescence signal	Without the need for EV pre-isolation; high sensitivity; rapid detection; low cost		[175]
CRISPR/Cas-assisted detection	Plasma	500 μL	miRNA-21; miRNA-221; miRNA-222	CRISPR/Cas9	High sensitivity and specificity		[176]

*PGR* Progesterone receptor, *ESR1* Estrogen receptor 1, *ERBB2* erb-b2 receptor tyrosine kinase 2, *PCAT6* Prostate cancer associated transcript 6

## New nucleic acids detection technologies

### ddPCR

Droplet digital PCR (ddPCR) is a technique in which the PCR reaction mixture is divided into tens of thousands of aqueous droplets in an oil emulsion (Fig. 5F). Each single droplet contains no more than one copy of target gene and is labeled as positive or negative according to the fluorescence amplitude. The concentration of target nucleic acids is then estimated by the Poisson distribution and ratio of the positive droplets [177]. The comparison between ddPCR and qPCR showed that ddPCR had higher accuracy and sensitivity in analyzing urinary exosomal miRNAs [163]. Chen et al. used ddPCR to identify IDH1 (isocitrate dehydrogenase 1) transcripts in exosomes derived from serum or cerebrospinal fluid (CSF) of glioblastoma (GBM) patients. Mutant *IDH1* mRNA was identified in CSF-derived exosomes of patients bearing mutant *IDH1* glioblastoma and higher level of *IDH1* mRNA was found in exosomes from patients with tumors than healthy controls [164]. Sun et al. recently used ddPCR to quantify 10 HCC-specific mRNA from plasma samples of HCC patients (Fig. 5F_a_). The diagnostic value of HCC exosome-derived mRNA signatures was evaluated by computing the digital scoring [61]. Moreover, exosomal miR-15a-5p was detected by ddPCR to distinguish endometrial cancer (EC) patients from healthy subjects [40]. Shen et al. reported that more copy numbers of lncRNAs RP11-77G23.5 and PHEX-AS1 were quantified by ddPCR in EpCAM-specific exosomes from malignant lung cancer patients compared to benign lung tumors [178].

### Molecular beacons

Molecular beacon (MB) is a hairpin-like oligonucleotide labeled with fluorescent dye and quencher at two ends of the probe (Fig. 5G). MB is designed to spontaneously hybridize with the targeted sequence, thereby destroying the hairpin ring structure and inducing the appearance of fluorescence [179]. Lee et al. observed that high fluorescent signals were obtained by the hybridization of molecular beacons and miRNA-21 in exosomes of cancer cells and human serum [122] (Fig. 5G_b_). Moreover, miR-375 and miR-574-3p were detected from exosomes of human urine by MB-based biosensors [168]. The expression levels of miR-21, miR-375, and miR-27a were detected in exosomes of human serum [121] (Fig. 5G_a_). Oliveira et al. utilized CPP (cell-penetrating peptides) to deliver MB across the plasma membrane and then to detect miRNA-451a in red blood cell (RBC)-derived exosomes [170]. Chen et al. designed a 2′-O-methyl and phosphorothioate modified molecular beacon to quantitatively analyze exosomal miRNA-1246 from human plasma. After rupturing the exosome membrane with Triton X-100, the probe could specifically target miRNA-1246 inside and display quantitative fluorescence signals [169]. Zhang et al. developed an integrated exosome isolation and detection system, in which exosomes could be separated by microfluidic technology via using a little volume of samples. Meanwhile, nanopore detection technology effectively improved the detection efficiency of tumor-related miRNA without the need for amplification and fluorescence labeling of detected objects [180]. The molecular beacon-based biosensor has the priority of simple procedure, free from exosome pre-isolation and nucleic acid extraction, indicating their great potential in liquid biopsy for cancer diagnosis and prognosis.

### DNA tetrahedron probe

DNA tetrahedron, a DNA nanoarchitecture with high controllability, can provide diverse amplified signal tags through chemical modification and DNA self-assembly [181]. Gao et al. conducted a DNA tetrahedron nanoprobe-based FRET sensing platform to sensitively detect miR-146b-5p in different cell lines [182]. Chen et al. reported a hairpin-tetrahedron nanoprobe for quantitative measurement of exosomal miR-21 in human serum [171]. In the absence of miR-21, the construction of fluorophore donor-quencher pairs resulted in a low FRET effect, while the presence of target miRNA induced the damage of stem-loop structure and occurrence of strong FRET. Consequently, the assay obtained a good linearity in the range from 1 × 10^− 12^ to 10 × 10^− 9^ M (mol/L) and a detection limit of 45.4 × 10^− 15^ M. Zhang et al. proposed a similarly electrochemical biosensor based on two multifunctional DNA tetrahedrons assisted catalytic hairpin assembly. By leverage localized reaction and cascade amplification, the sensor enabled sensitive detection of tumor-associated exo-miRNAs down to 7.2 aM in 30 min [172].

### Localized surface plasmon resonance

Localized surface plasmon resonance (LSPR) occurs when the incident photon frequency matches the overall vibration frequency of precious metal nanoparticles or metal conducting electrons (Fig. 5H). Joshi et al. developed a biosensor based on LSPR for the label-free and nondestructive measurement of exosomal miR-10b [173]. Gold nanoprisms were chemically synthesized onto a silanized glass substrate and then functionalized with HS-C6-ssDNA and PEG6-SH. The direct hybridization of target miR-10b and HS-C6-ssDNA induced the formation of double-strand DNA which could increase the index of refraction of nanoprisms and change the wavelength of LSPR dipole peak (λLSPR). The concentration of miR-10b could be evaluated by ΔλLSPR. This platform was sensitive enough to distinguish between miR-10b and miR-10a (only one nucleotide difference) even in the subattomolar concentration range. Wu et al. developed an SPRi-based biosensor to detect multiple exosomal miRNAs for accurate diagnosis of NSCLC, in which Au-on-Ag heterostructure and DNA tetrahedral framework were utilized to enhance SPR signal and each exosomal miRNA could be ultrasensitively identified with different SPR signals [123] (Fig. 5H_a_).

### TIRF-based single-vesicle imaging

As a single-exosome analytic method, total internal reflection fluorescence (TIRF) has emerged for providing additional information about the heterogeneity of exosomes (Fig. 5E). He et al. proposed a TIRF-based single-vesicle imaging assay which delivered molecular beacon probes into exosomes and thus induced an amplified fluorescence of target miRNA (Fig. 5E_b_). They performed a direct visualization of single vesicles and in-situ quantitative analysis of miR-21 in human serum samples and found that this assay showed better performance than conventional PCR assay in distinguishing cancer patients from healthy subjects [119].

### Thermophoresis-assisted detection

Zhao et al. developed a thermophoretic sensor for in situ detection of exosomal miRNAs without the need of RNA extraction or target amplification (Fig. 5F). Aptamer modified in nanoflares could bind to target exosomal miRNA, inducing the appearance of fluorescence. The fluorescent signal became amplified after thermal electrophoretic accumulation, allowing the sensitive detection of 0.36 fM exosomal miRNA in a small volume of serum sample [175]. In addition, Han et al. proposed a DNA tetrahedron-based thermophoretic assay (DTTA) which achieved a detection limit of 14 aM mRNA in serum exosomes. After internalized by exosomes, the two fluorophore-labeled recognition sequences of DNA tetrahedron could bind to target mRNA, leading to an increase in FRET signal. Thermophoretic effect was applied to further amplify the FRET signal, which achieved a highly sensitive detection of PSA mRNA in exosomes. The DTTA assay showed that exosomal PSA mRNA performed better than serum PSA protein in discriminating prostate cancer from benign prostatic hyperplasia (AUC: 0.93 versus 0.74), providing a new approach for precise detection of prostate cancer [183].

### CRISPR/Cas system-assisted detection

Recently, CRISPR/Cas system has offered new opportunities to develop analytical methods for the detection and quantification of nucleic acids in exosomes. The strategy of integrating nucleic acid amplification with CRISPR/Cas has been proposed to improve analytical specificity and sensitivity [184]. For instance, the platform of rolling circular amplification-assisted CRISPR/Cas9 cleavage (RACE) was conducted by Wang et al. to detect multiple exosomal miRNAs [176]. During RCA process, padlock probe recognized single base differences in the presence of HiFi Taq DNA ligase. The amplification product of long ssDNA, which consisted of a large number of repeated target sequences and PAM structures, was specifically recognized by Cas9 nuclease. Consequently, the TaqMan probe hybridized with ssDNA was completely cleaved by Cas9 protein, allowing “turn on” fluorescence change that could be conveniently measured by a spectroscopy. As a result, this RACE platform could be used for highly specific detection of single or multiple exosomal miRNAs from human plasma. However, the large number of naturally PAM structures in the genome may increase the risk of off-target effects, greatly impairing the specificity of analysis [185].

## Machine learning

Machine learning refers to a technology that predicts and analyzes unknown data by building models of known data. As a crucial branch of artificial intelligence, machine learning has been widely used for the multiplex profiling of exosomal biomarkers. Algorithms such as linear discriminant analysis (LDA), principal component analysis (PCA), neural network (NN), support vector machine (SVM), and random forests (RF) have been developed to classify multivariable data into a typical classification model, which are then applied for the prediction and grouping of unknown biological data [186]. Kawakami et al. demonstrated that machine learning algorithms performed better than traditional logistic regression analysis to predict and diagnose epithelial ovarian cancer [187]. Wu et al. used the fluorescent signal of urine-derived exosomes as input data, and KNN (K-Nearest Neightbors) and SVM serving as machine learning models, were applied for exosomal biomarker analysis. By introducing machine learning algorithm, the diagnostic model could make an accurate diagnosis and classification of multiple diseases [188]. Liu et al. used LDA to determine a sum signature of seven exosomal biomarkers, which achieved a high accuracy in discriminating prostate cancer from benign disease [68]. Tian et al. analyzed a weighted sum of eight cancer-associated proteins of exosomes from 1 μL plasma via LDA [69]. PCA is an algorithm that transforms a group of probably-related variables into a series of linearly-unrelated variables through orthogonal transformation. Shin et al. used PCA to explore the features of cell exosomes and human plasma exosomes. As a result, the machine learning model could classify normal and lung cancer cell lines-derived exosomes with an accuracy of 95% and obtain an AUC of 0.912 in predicting lung cancer for the whole cohort [162]. Liu et al. employed RF, NN, and SVM to analyze multiple exosome-derived mRNAs of breast cancer patients, which exhibited improved diagnostic performance compared to a single marker [120]. These studies suggest the great potential of combining exosome analysis and machine learning in cancer liquid biopsy. Notably, large data sets are required for typical machine learning approaches, while a limited number of clinical samples and insufficient data may lead to poor accuracy and reliability. The emergence of more powerful machine learning algorithms will favor the analysis of exosomes for liquid biopsy.

## The implication of exosomes in cancer liquid biopsy

**Fig. 6:**
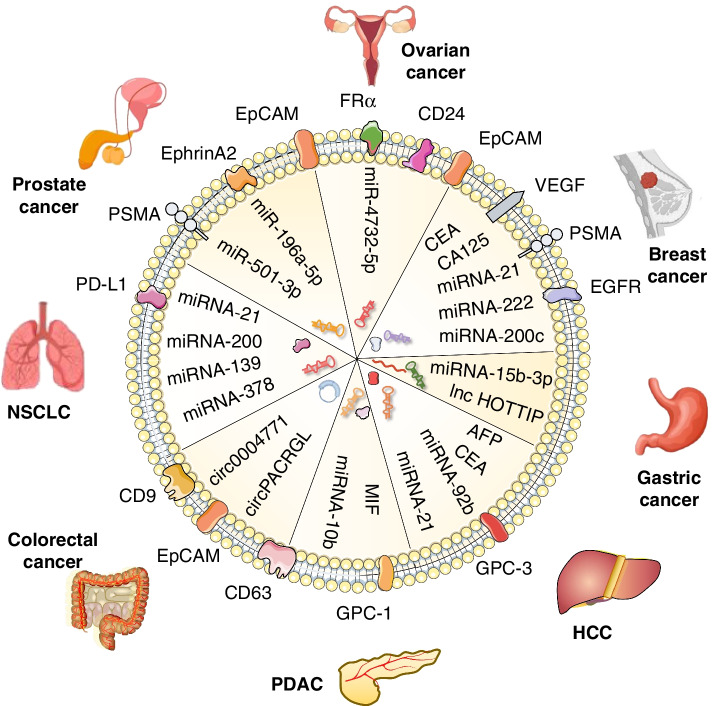
The application of exosomes in cancer liquid biopsy. Cancer-derived exosomes are enriched in differentially expressed proteins and nucleic acids, and have been tested as new biomarkers for the early diagnosis, stage classification, and prognosis prediction of different cancers, highlighting their important value in cancer liquid biopsy and precision medicine

Recent studies have shown that exosomes are superior to CTCs and ctDNA in liquid biopsy for early diagnosis, disease monitoring, and prognosis prediction [34, 189, 190]. Herein, we summarized the application of exosomes in liquid biopsy for various cancers (Fig. 6, Table 4).

**Table 4:** Exosomes as biomarkers for cancer liquid biopsy

Cancer types	Exosome sources	Sample volume	Exosomal biomarker	Clinical samples	Diagnostic performance	Clinical significance	Ref.
Gastric Cancer	Serum		lnc HOTTIP	126 GC patients; 120 healthy donors	AUC = 0.827	Early diagnosis	[191]
	Serum		miR-15b-3p	108 GC patients; 108 healthy donors	AUC of 0.820; specificity of 80.6%; sensitivity of 74.1%	Early diagnosis	[192]
HCC	Plasma	100 μL	AFP; GPC3; ALB; APOH; FABP1; FGB; FGG; AHSG; RBP4; TF mRNA	36 HCC patients; 26 Cirrhosis	AUC of 0.87; sensitivity of 93.8%; specificity of 74.5%	Early diagnosis	[61]
	Serum	500 μL	miRNA-21; lncRNA-A TB	72 HCC patients	Higher in HCC patients	Prognostic significance	[189]
	Serum		miR-21		Higher in HCC patients	Early diagnosis	[193]
	Serum	250 μL	miR-92b	28 non-HCC; 28 HCC patients without recurrence; 43 HCC patients with early recurrenc	Sensitivity of 85.7%; specificity of 86.0%; AUC = 0.925	Early recurrence diagnosis after LDLT	[194]
	Serum	100 μL	CEA; GPC-3 and PD-L1	12 HCC patients; 12 hepatitis B; 6 healthy donors	Higher in HCC patients	Early diagnosis and progression monitoring	[112]
PDAC	Plasma	500 μL	miRNA-10b	3 PDAC Patients; 3 CP Patients; 3 healthy donors	Higher in PDAC patients	Early diagnosis and progression monitoring	[173]
	Plasma		miRNA-10b	PDAC patients; CP patients and healthy donors	Higher in PDAC patients	Early diagnosis	[195]
	Mouse plasma samples		miR-3970-5p	9 healthy donors; 9 PanIN patients; 9 PDAC patients	Accuracy of 65%	Early diagnosis	[196]
	Serum	250 μL	Glypican1	192 patients; 100 healthy donors	Sensitivity of 100%; specificity of 100%; positive predictive value of 100%; negative predictive value of 100%; AUC of 1.0	Early diagnosis	[38]
	Plasma	25 μL	Glypican1	20 PDAC patients; 7 benign pancreatic disease; 11 healthy donors	99% sensitivity and 82% specificity	Stage classification	[197]
	Serum	5 μL	EpCAM, Glypican1		90% accuracy for pancreatic cancer or normal pancreatic epithelial cell lines; 87 and 90% predictive accuracy for HC and EPC individual samples	Early diagnosis	[198]
	Serum	2 μL	MIF	4 patients at stage 1 ~ 2; 37 patients at stage 3	Discriminatory sensitivity of 95.7%	Stage classification	[199]
CRC	Serum		hsa-circ-0004771	179 patients; 45 healthy donors	AUC of 0.86, 0.88 to differentiate stage I/II CRC patients and CRC patients from HCs	Early diagnosis	[200]
	Plasma	25 μL	Epcam-CD63	59 cancer patients; 20 healthy donors	AUC of 0.96	Early diagnosis; prognosis prediction	[125]
NSCLC	Plasma		miRNA-21; miRNA-139; miRNA-200; miRNA-378	5 patients; 5 healthy donors	Higher in NSCLC patients	Early diagnosis	[123]
	Plasma	1 mL	miRNA-21	NSCLC patients; recurrence of NSCLC patients; healthy individuals	Higher in NSCLC patients	Early diagnosis and drug resistance in advanced cancers	[201]
	Plasma	1.5 mL	CD63; EGFR; EpCAM	4 patients; 4 treated patients; 4 healthy donors	Higher in NSCLC patients	Early diagnosis and therapeutic effect evaluation	[159]
	Serum	50 μL	PD-L1	5 patients; 4 healthy donors	Higher in NSCLC patients	Early diagnosis	[132]
	Serum	4 μL	PD-L1	7 patients at stage 1 ~ 2; 10 patients at stage 3 ~ 4; 12 healthy controls	AUC of 0.97	Early diagnosis	[90]
Breast Cancer	Plasma		EpCAM	6 BC patients; 3 healthy donors	Higher in BC patients	Early diagnosis	[71]
	Plasma		EpCAM; HER2	10 BC patients; 5 healthy donors	AUC of 1; AUC of 1	Early diagnosis	[134]
	Serum	3.6 μL	EpCAM	20 BC patients; 10 healthy donors	AUC _BC versus HD_ = 0.99; AUC _HER2+ BC versus HER2– BC_ = 0.94	Cancer classification	[202]
	Serum		PD-L1	7 patients with metastatic; 8 patients without metastatic; 6 healthy donors	Higher in BC patients	Prognosis prediction and progression monitoring	[114]
	Blood		CA153	104 BC patients; 100 breast hyperplasia patients and 100 healthy controls	Higher in BC patients	Differential diagnosis	[203]
	Serum		miR-21; miR-222; miR-200c	Luminal, HER2^+^, and TN breast cancer patients	Higher in BC patients	Classification of molecular subtypes of breast cancer	[204]
	Plasma	1 μL	CA153; EpCAM	36 MBC patients before salvage treatment; 21 NMBC patients before surgical therapy; 66 age-matched healthy donors	AUPRC _CA153_ = 0.9286	Differential diagnosis of BC and healthy donors	[69]
					AUPRC _EpCAM_ = 0.9709		
	Plasma	1 μL	CA153; CA125; CEA; HER2; EGFR; PSMA; EpCAM; VEG	36 MBC patients before salvage treatment; 21 NMBC patients before surgical therapy; 66 age-matched healthy donors	AUPRC of 0.9826	Differential diagnosis of BC and healthy donors	[69]
	Plasma	1 μL	CA153; CA125; CEA; HER2; EGFR; PSMA; EpCAM; VEG	36 MBC patients before salvage treatment; 21 NMBC patients before surgical therapy; 66 age-matched healthy donors	AUPRC of 0.8672	Differential diagnosis of MBC and NMBC	[69]
	Plasma samples		EpCAM	Various breast cancer patients and healthy individuals	Higher in BC patients	Early diagnosis	[115]
Prostate Cancer	Urine	50-150 mL	miR-196a; miR-143-3p; miR-196-5p; miR-501-3p;	28 PCA patients; 19 healthy donors	AUC _miR-196a_ = 0.92	Early diagnosis	[205]
					AUC _miR143-3p_ = 0.72		
					AUC _miR196-5p_ = 0.73		
					AUC _miR501-3p_ = 0.69		
	Plasma	750 μL	miR-217; miR-23b-3p	10 patients; 10 healthy donors	Higher in PCA patients	Early diagnosis	[206]
	Serum	400 μL	EphrinA2	50 PCA patients; 21 BPH patients; 20 healthy donors	AUC of 0.7666	Early diagnosis; distinguish PCA from BPH patients	[207]
	Serum	25 μL	EpCAM and PSMA	10 PCA patients; 5 healthy donors	Higher in PCA patients	Early diagnosis	[127]
	Serum		TUBB3 mRNA	52 mCRPC patients	Higher in PCA patients	Prognosis	[208]
Ovarian Cancer	Ascites		EpCAM; CD24	20 patients; 10 healthy donors	Higher in OVCA patients	Early diagnosis	[116]
	Plasma	2 mL	CA125; EpCAM; CD24	15 patients; 5 healthy donors	AUC _CA125_ = 1.0	Early diagnosis	[74]
					AUC _EpCAM_ = 1.0		
					AUC _CD24_ = 0.91		
	Plasma	20 μL	CD24; EpCAM; FRα	20 OVCA patients; 10 non-cancer controls	AUC _CD24_ = 1.0	Early diagnosis	[165]
					AUC _EpCAM_ = 1.0		
					AUC _FRα_ = 0.995		
	Plasma	200 μL	miR-4732-5p	21 healthy controls and 34 epithelial ovarian cancer patients	AUC _miR-4732-5p_ = 0.889	Early diagnosis	[209]

*ALB* Albumin, *APOH* Apolipoprotein H, *AUPRC* Area under the Precision-Recall Curves, *FABP1* Fatty acid binding protein 1, *FGB* Fibrinogen beta chain, *FGG* Fibrinogen gamma chain, *AHSG* Alpha 2-HS glycoprotein, *RBP4* Retinol binding protein 4, *TF* Transferrin, *LDLT* Living donor liver transplantation, *CP* Chronic pancreatitis, *PanIN* Pancreatic intraepithelial neoplasia, *PanIN* Pancreatic intraepithelial neoplasia, *MIF* Migration inhibitory factor

### HCC

Hepatocellular cancer is the fourth cause of cancer-related death. Molecular signatures loaded into HCC-derived exosomes may be used to for diagnosis. Exosomal miR-21 was found to suppress the apoptosis of HCC cells and be upregulated in HCC patients [112]. Exosomal proteins such as CEA and GPC-3, were able to distinguish HCC patients from healthy subjects [193], which may serve as promising biomarkers for noninvasive cancer diagnosis. In a study containing 158 samples, plasma was collected for the detection of 10 HCC-specific genes, including alpha-fetoprotein (AFP), GPC3, albumin, apolipoprotein H, etc. [61]. It was observed that higher fluorescent signals were obtained in HCC cohort via ddPCR in comparison with noncancer cohorts. The 10 gene signatures were further computed by machine learning, which showed great potential to distinguish early-stage HCC from at-risk liver cirrhosis with an AUC of 0.93. Nakano et al. compared the expression levels of exosomal miR-92b and circulating AFP among HCC patients who received liver transplantation [194]. It was revealed that exosomal miR-92b could predict early recurrence of HCC with an AUC of 0.925, while the AUC of AFP was 0.651. Zhu et al. integrated plasma exosomal RNA-sequence, cell-free RNA-sequence and TCGA tissue RNA-sequence datasets to identify 5 noncoding RNAs (circ-0073052, circ-0080695, SNORD3B-1, LINC01226 and HULC) as potential biomarkers of liver cancer. In addition, a panel of SNORD3B-1, circ-0080695 and miR-122 showed the highest AUC (89.4%) to classify liver cancer patients from healthy donors in comparison with other marker panels. By the selected panel, 79.2% AFP-negative samples and 77.1% early-stage liver cancer samples were successfully detected in the testing and validation sets, which indicated the potential of exRNAs panel in the early diagnosis of liver cancer [210].

### PDAC

As one of the most recognized indicators associated with pancreatic cancer progression, miRNA-10b is now being widely studied for the early diagnosis of pancreatic ductal adenocarcinoma (PDAC) [211]. Joshi et al. indicated that the levels of exosomal miR-10b were remarkably different among pancreatic cancer patients, at-risk patients with chronic pancreatitis (CP) and healthy individuals, which suggested the potential of miR-10b in diagnosing pancreatic cancer and predicting CP patients who may develop to PDAC [173]. The same conclusion was obtained by Pang’s group by using a dual-SERS biosensor for one-step detection of mRNAs in exosomes [195]. Melo et al. found that GPC-1^+^ circulating exosomes (GPC-1^+^ crExos) exhibited high specificity and sensitivity to identify PDAC patients from healthy individuals and chronic pancreatitis (AUC = 1.0), which was superior to CA199 (AUC = 0.739). Moreover, the levels of GPC-1^+^ crExos were correlated with tumor burden and survival in patients before and after surgery, suggesting the potential of GPC-1 serving as a reliable biomarker for treatment effect and prognostic monitoring [38]. In addition, the combination of GPC-1 and CD63 was demonstrated to show 99% sensitivity and 82% specificity in distinguishing PDAC patients from healthy subjects [197]. Furthermore, migration inhibitory factor (MIF) was reported to have better performance than GPC-1 and EGFR in the discrimination of different stages of pancreatic cancer [199].

### CRC

Colorectal cancer (CRC) is one of the most common malignancies with high morbidity and mortality [1]. CircRNA in exosomes is related to the occurrence and development of cancer. Due to the circular structure, it provides a stable biomarker for cancer diagnosis. CircRNA-0004771 was found to be upregulated in serum exosomes of CRC patients [200]. The AUC of exosomal circ-0004771 to identify CRC from healthy controls was 0.88, and that to distinguish patients with stage I and II from other benign intestinal diseases was 0.816, which indicated that circ0004771 could be used as a potential diagnostic marker for colorectal cancer. Wei et al. explored novel exosome biomarkers for the early diagnosis and prognosis of colorectal cancer [125]. The detection of plasma samples revealed that the expression of CD9, CD63 and EpCAM were significantly higher in CRC patients compared with healthy and benign controls (with an AUC of 0.90 and 0.96, respectively). In addition, miR-15b, miR-21, and miR-31 were reported to be highly expressed in serum exosomes of CRC patients. ROC curves showed that AUC of miR-15b was 0.86 and the combined miR-15b, miR-21, and miR-31 panel exhibited 81.21% sensitivity and 81.03% specificity [212].

### NSCLC

Non-small cell lung cancer accounts for over 80% of lung cancer-induced deaths [1]. High level of PD-L1 was detected in serum exosomes of NSCLC patients compared to normal individuals [132]. The diagnostic value of exosomal PD-L1 was further confirmed by Pang et al. in a larger cohort of patients with an AUC of 0.97 [90]. Ma et al. found that the expression of exosomal miR-21 was higher in patients with recurrent NSCLC compared with healthy individuals [201]. However, high levels of exosomal miR-21 were also identified in other cancers such as colorectal cancer, breast cancer, and liver cancer [123, 189, 204]. Therefore, the detection of multiple miRNAs may be more valuable for NSCLC diagnosis. Wu et al. demonstrated that miR-21, miR-378, miR-139, and miR-200 were differentially expressed between NSCLC patients and healthy donors, providing more optional biomarkers for the early diagnosis of NSCLC [123]. Notably, the experimental results showed that more amounts of exosomal RNAs were obtained in urine-derived exosomes of NSCLC patients than that from plasma and bronchoalveolar lavage fluid, which may provide a new direction of sample selection to analyze exosomes [62].

### Breast cancer

Breast cancer (BC) is the most common cause of cancer-related death among females. Early diagnosis can effectively suppress the mortality and achieve better treatment outcomes. Despite as a widely used biomarker, CA15-3 (carbohydrate antigen 15-3) is not sensitive enough to diagnose primary and metastatic breast cancer (MBC) [203]. Lee et al. demonstrated that cancer cell-derived exosomal miR-21, miR-222, and miR-200c could be quantified in body fluids and used for breast cancer diagnosis [204]. Lu et al. reported that the combination of RDW (red blood cell distribution width), MPV (mean platelet volume), and CA15-3 showed better specificity and sensitivity to identify breast cancer than single biomarker [203]. In addition, an exosome signature was identified by computing weighted sum of eight biomarkers (CA15-3, CA125, CEA, HER2, EGFR, PSMA, EpCAM, and VEGF) through machine learning. Consequently, the signature showed a high discriminative accuracy to differentiate MBC from non-metastatic ones and age-matched healthy donors (91.1%). Moreover, the exosome signature was reported to accurately monitor MBC treatment response and serve as an independent prognostic factor for progression-free survival in MBC patients.

### Prostate cancer

PSA is a widely used biomarker for the detection of prostate cancer. However, increased PSA was also identified in inflammatory diseases such as benign prostatic hyperplasia (BPH) [213]. Therefore, the development of sensitive and specific biomarker is urgently needed to diagnose patients at early stage and monitor the progression of cancer. Li et al. reported that exosomal ephrinA2 had superior capability to blood circulating PSA to differentiate prostate cancer patients from BPH patients with an AUC of 0.906 [207]. Moreover, increased EpCAM and PSMA were found in the serum exosomes of prostate cancer [127]. In addition, the expression of exosomal TUBB3 mRNA was reported to be associated with poor progression-free survival of abiraterone in metastatic castration-resistant prostate cancer patients [208].

### Exosomes in clinical trial and use for cancer liquid biopsy

Due to the priority of living-cell secretion, large amounts and stable circulation compared to CTC and ctDNA, exosome-based liquid biopsy has been tested in clinical trials and several of them have been approved and reached the market. In 2016, *Exosome Diagnostics* proposed the first exosome-based liquid biopsy in the world, ExoDx™ Lung (ALK), for the isolation and analysis of exosomal RNA from blood samples. At CLIA-certified laboratory, ExoDx™ Lung (ALK) was proved to be an accurate, real-time tool to detect EML4-ALK mutations in NSCLC patients with 88% sensitivity and 100% specificity, which provides a more direct and sensitive method to detect gene fusions than cfDNA. In addition, the ExoDx Prostate IntelliScore (EPI) has been certified by FDA. Based on the detection of ERG, PCA3, and SPDEF RNA in exosomes, EPI provides a risk score to predict whether a patient with PSA from 2 to 10 ng/mL is likely to develop higher-grade prostate cancer [214]. According to ExoDx, 93% of sensitivity was achieved in prospective studies, and 26% of unnecessary needle biopsies were avoided when the EPI threshold was set at 15.6 [215]. Three independent, prospective, and multicenter clinical trials declared that EPI outperformed standard of care and could be used to assist in the early diagnosis of prostate cancer and eliminate unnecessary prostate biopsy [64]. Moreover, MedOncAlyzer 170 is a newly developed liquid biopsy system capable of detecting both exosomal RNA and ctDNA in a single trial. It can identify significant and functional mutations in multiple cancer types from small volumes (0.5 ml) of patient blood or plasma. Due to the unique formation manner of exosome and ctDNA, MedOncAlyzer 170 is accurate and highly sensitive to detect mutation at all stages of cancer progression and treatment. Although the clinical application value has been verified, larger clinical samples, populations and trials are still needed to confirm the role of exosome-based liquid biopsy in cancer diagnosis and treatment.

## Conclusions and perspectives

At present, the limitation of tissue biopsy has been gradually recognized in the field of precision medicine. On the contrary, liquid biopsy has the advantages of minimal invasiveness, easy sample acquisition, and dynamic analysis. Exosomes have been confirmed to stably circulate in body fluids and contain diverse information that reflects the status of tumor progression [28, 33]. The potential of exosome serving as diagnostic and prognostic biomarkers has been investigated in a variety of cancers. However, the high heterogeneity and nano-size of exosomes have posed great technical challenges to the acquisition of their molecular information and interactions. In this review, we have summarized the advantages and drawbacks of conventional and novel techniques to isolate, characterize and detect exosomes. Techniques based on physical or biological characteristics are being widely developed for exosome separation, and the application of microfluidic devices holds great potential for the ultrafast separation of pure exosomes with high yields. Although revolutionary progress has been achieved, there is no standardized method for the high-throughput, high-purity, and minimal damage separation of exosomes from both cell culture medium and human body fluids. Diverse molecules contained in the circulating exosomes have highlighted the potential of exosomes in liquid biopsy. Faster and more convenient methods are required to validate the exosomal cargoes as biomarkers in the diagnosis of cancer. Most new detection platforms, although superior to conventional methods, still face the challenge of low sensitivity and high heterogeneity of different exosome subsets. The technology of single exosome detection and analysis may reveal the unique molecular profile of specific exosomes and provide a plausible strategy to obtain accurate cancer-related information. The further exploration in exosome heterogeneity will address many of the challenges in current exosome studies. Improvements in developing new strategies to isolate exosomes from body fluids and profile exosomal contents in a fast and sensitive way will facilitate the practical application of exosome-based liquid biopsy for cancer precision medicine.

## Data Availability

Not applicable.
